# Accurate cell type annotation for single‐cell chromatin accessibility data via contrastive learning and reference guidance

**DOI:** 10.1002/qub2.33

**Published:** 2024-02-08

**Authors:** Siyu Li, Songming Tang, Yunchang Wang, Sijie Li, Yuhang Jia, Shengquan Chen

**Affiliations:** ^1^ School of Statistics and Data Science Nankai University Tianjin China; ^2^ School of Mathematical Sciences and LPMC Nankai University Tianjin China

**Keywords:** cell type annotation, chromatin accessibility, novel type, reference‐guided, single‐cell

## Abstract

Recent advances in single‐cell chromatin accessibility sequencing (scCAS) technologies have resulted in new insights into the characterization of epigenomic heterogeneity and have increased the need for automatic cell type annotation. However, existing automatic annotation methods for scCAS data fail to incorporate the reference data and neglect novel cell types, which only exist in a test set. Here, we propose RAINBOW, a reference‐guided automatic annotation method based on the contrastive learning framework, which is capable of effectively identifying novel cell types in a test set. By utilizing contrastive learning and incorporating reference data, RAINBOW can effectively characterize the heterogeneity of cell types, thereby facilitating more accurate annotation. With extensive experiments on multiple scCAS datasets, we show the advantages of RAINBOW over state‐of‐the‐art methods in known and novel cell type annotation. We also verify the effectiveness of incorporating reference data during the training process. In addition, we demonstrate the robustness of RAINBOW to data sparsity and number of cell types. Furthermore, RAINBOW provides superior performance in newly sequenced data and can reveal biological implication in downstream analyses. All the results demonstrate the superior performance of RAINBOW in cell type annotation for scCAS data. We anticipate that RAINBOW will offer essential guidance and great assistance in scCAS data analysis. The source codes are available at the GitHub website (BioX‐NKU/RAINBOW).

## 1 INTRODUCTION

Chromatin accessibility, a fundamental property of DNA that plays a critical role in gene regulation and cell identity, refers to the degree that nuclear macromolecules can access and interact with DNA [1, 2]. With the rapid advances in single‐cell chromatin accessibility sequencing (scCAS) technologies, the importance of cell type annotation in scCAS data is on the rise due to its potential to capture the chromatin regulatory landscape that controls gene transcription in each cell type [3, 4, 5, 6]. Moreover, the identification of novel cell types during cell type annotation has significant implications, as it offers the potential to reveal previously undiscovered biological processes and cellular functions [7, 8, 9].

Currently, there are two main approaches for annotating cell types. The first approach involves the unsupervised clustering of cells and assigning labels to each cluster based on cluster‐specific marker genes [10, 11]. However, the manual method is non‐reproducible and increasingly challenging when larger numbers of individual cells are being analyzed. Another approach to cell type annotation utilizes the annotated training data to automatically label cells in scCAS data. Some classic machine learning methods used for single‐cell RNA sequencing annotation, such as support vector machine (SVM) [12], random forest (RF) [13], and K nearest neighbors (KNN) [14] can be applied to the automatic annotation task of scCAS data [4]. Recently, a probabilistic model named EpiAnno has been proposed, which is the first automatic annotation method specifically for scCAS data [4]. However, there are still significant limitations of existing methods in the automatic annotation task as follows. Firstly, existing methods cannot achieve accurate annotation due to the extreme sparsity and the close‐to‐binary nature of scCAS data [3]. Secondly, the extensive availability of bulk chromatin accessibility data [15, 16, 17] and the accumulating scCAS data [1, 17, 18, 19, 20] provide abundant opportunities to incorporate reference data during the training process, and the reference‐guided approach has been successfully applied to various analyses of single‐cell omics data [21, 22, 23, 24]. However, current automatic annotation methods for scCAS data fail to utilize the reference data information. Thirdly, the discovery of new disease biomarkers and drug targets hinges on the accurate identification of novel cell types [7, 8, 9], whose existence in test sets may not have been previously represented in the training set. Despite the importance, current computational methods neglect the issue of novel cell types in test sets, posing a challenge to fully understanding cellular diversity.

To fill these gaps, we propose RAINBOW, a **r**eference‐guided **a**utomat**i**c a**n**notation method, which is **b**ased on the c**o**ntrastive learning frame**w**ork and can effectively identify novel cell types in the test set. More specifically, we construct a cell type automatic annotation model based on the contrastive learning framework [25, 26, 27], which focuses on learning common features among cells of the same type and on distinguishing differences between non‐similar cells, thereby enhancing knowledge of the heterogeneity of different cell types. Additionally, we incorporate information from external reference data as prior knowledge, intending to achieve more accurate annotation. Furthermore, through unsupervised clustering of unlabeled data and by selecting the clusters with higher average entropy, RAINBOW can identify novel cell types in a test set effectively.

With comprehensive experiments on multiple scCAS datasets, we demonstrated that RAINBOW outperformed other existing methods in annotating known cell types. In addition, we verified that RAINBOW offered superior performance from various perspectives. On one hand, with changes in sparsity and the number of cell types, RAINBOW exhibited more stability compared to other methods. On the other hand, RAINBOW accurately annotated cells in the inter‐dataset annotation and had the potential to reveal cell type‐specific motifs. We also investigated the improvement of incorporating reference data by comparison of the annotation performance with that for which no reference data is incorporated. Moreover, through a series of experiments, we verified the effectiveness of RAINBOW in identification of novel cell types, which is expected to provide great assistance to the discovery of new disease biomarkers.

## 2 RESULTS

### 2.1 The overview of RAINBOW

**FIGURE 1:**
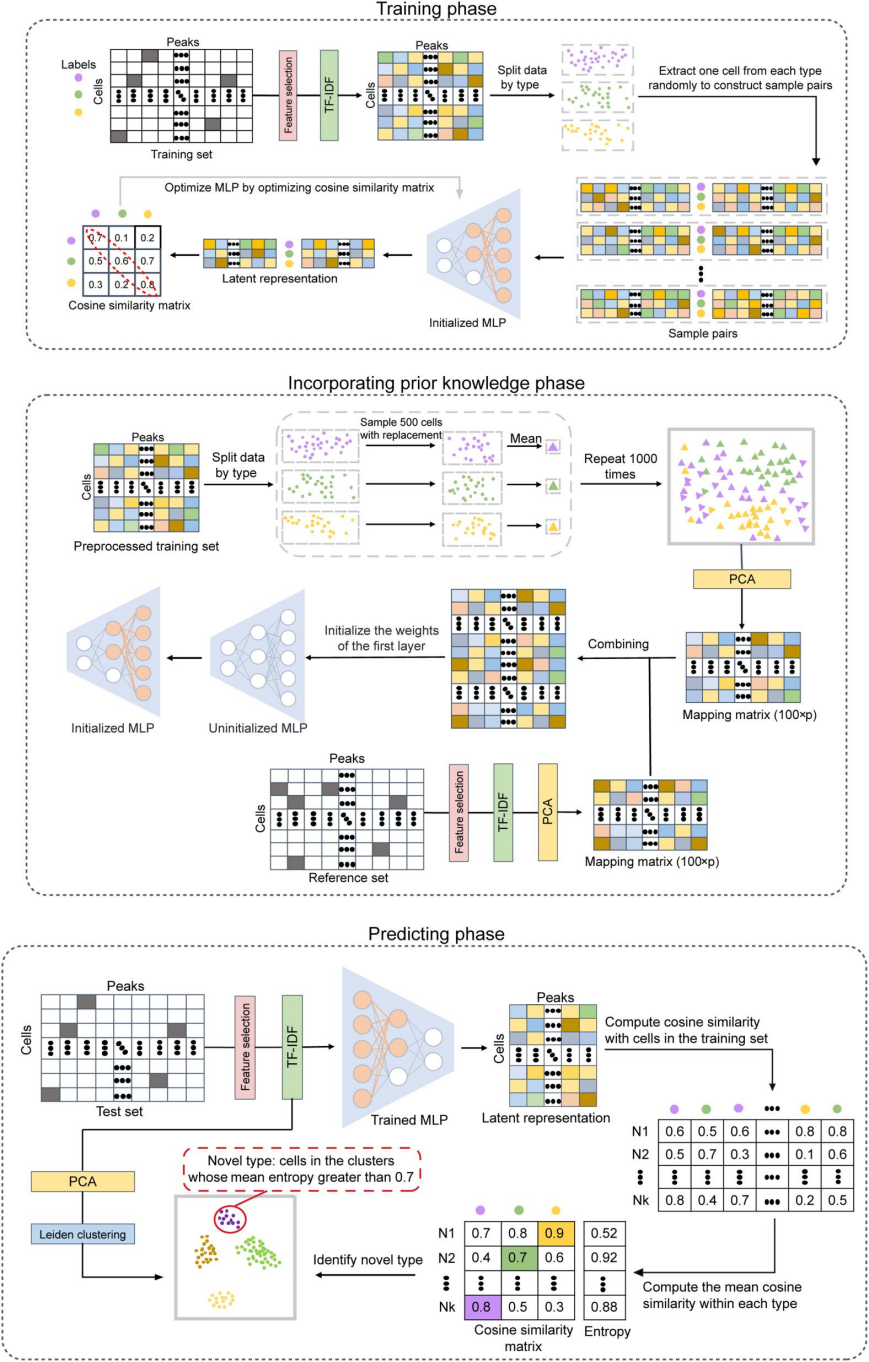
The framework of RAINBOW.

As shown in Figure 1, the framework of RAINBOW has three main modules: training phase, phase of incorporating prior knowledge, and prediction phase.

During the training phase, after feature selection and term frequency‐inverse document frequency (TF‐IDF) transformation (see more details in Section 5), we utilize contrastive learning to learn latent representations of the training set, which have yielded state‐of‐the‐art results on various tasks in single‐cell data analysis [28, 29, 30]. Specifically, we utilize the multi‐layer perceptron (MLP) module within the contrastive learning framework, as suggested by recent studies [31, 32, 33]. By optimizing the MLP based on the contrastive learning strategy, we can obtain the effective low‐dimensional representations (see more details in Section 5).

At the stage of incorporating reference data, we provide two options in this phase: either, utilizing the information from external reference data, or, only utilizing the information from the training set (see more details in Section 5). Specifically, we incorporate information from the training set or reference set by initializing the weights connecting the first to the second layer of MLP using the weights of principal component analysis (PCA), since both the first layer of a neural network and PCA can be understood as dimensionality reduction techniques. The effectiveness of this initialization strategy has been validated in several studies [21, 22, 23, 24].

In the prediction phase, we use the trained MLP to obtain the latent representation and predict cell types based on the cosine similarity (see more details in Section 5). Notably, RAINBOW is capable of identifying novel cell types effectively. We apply Leiden clustering [34] to the latent representation of spots obtained from PCA and annotate cells in clusters with a mean entropy value >0.7 as the novel type. The threshold of 0.7 is recommended by a benchmark study [35].

### 2.2 RAINBOW enables accurate annotation on scCAS data

To validate the superior performance of RAINBOW on scCAS datasets, we firstly conducted five‐fold cross‐validation on each of the eight scCAS datasets of BoneMarrowA [18], BoneMarrowB [18], donorBM0828 [1], Melanoma [36], common lymphoid progenitor (CLP)/lymphoid‐primed multipotent progenitor (LMPP)/multipotent progenitor (MPP) [1], HematopoieticCells [1], Forebrain [20] and Cerebellum [18], which were profiled from different species and tissues and had different degrees of imbalance. In detail, we randomly divided all cells into five folds and performed a series of iterations where we trained a cell type annotation model using four of the folds and used the trained model to predict the cell type labels in the remaining fold. As suggested in the recent benchmark study [35], we used EpiAnno [4], SVM [12], RF [13], and KNN [14] (with K values of 9 and 50) as baseline methods (see more details in Section 5). Specifically, to eliminate potential bias, we only utilized the information of the training set in the experiment and did not incorporate external reference data since the baseline methods did not possess the ability to incorporate reference data. Moreover, we benchmarked the annotation performance using three metrics, macro‐F1 score, Cohen’s kappa value (Kappa) [37] and Jaccard, as suggested by recent studies [4, 31, 38].

**FIGURE 2:**
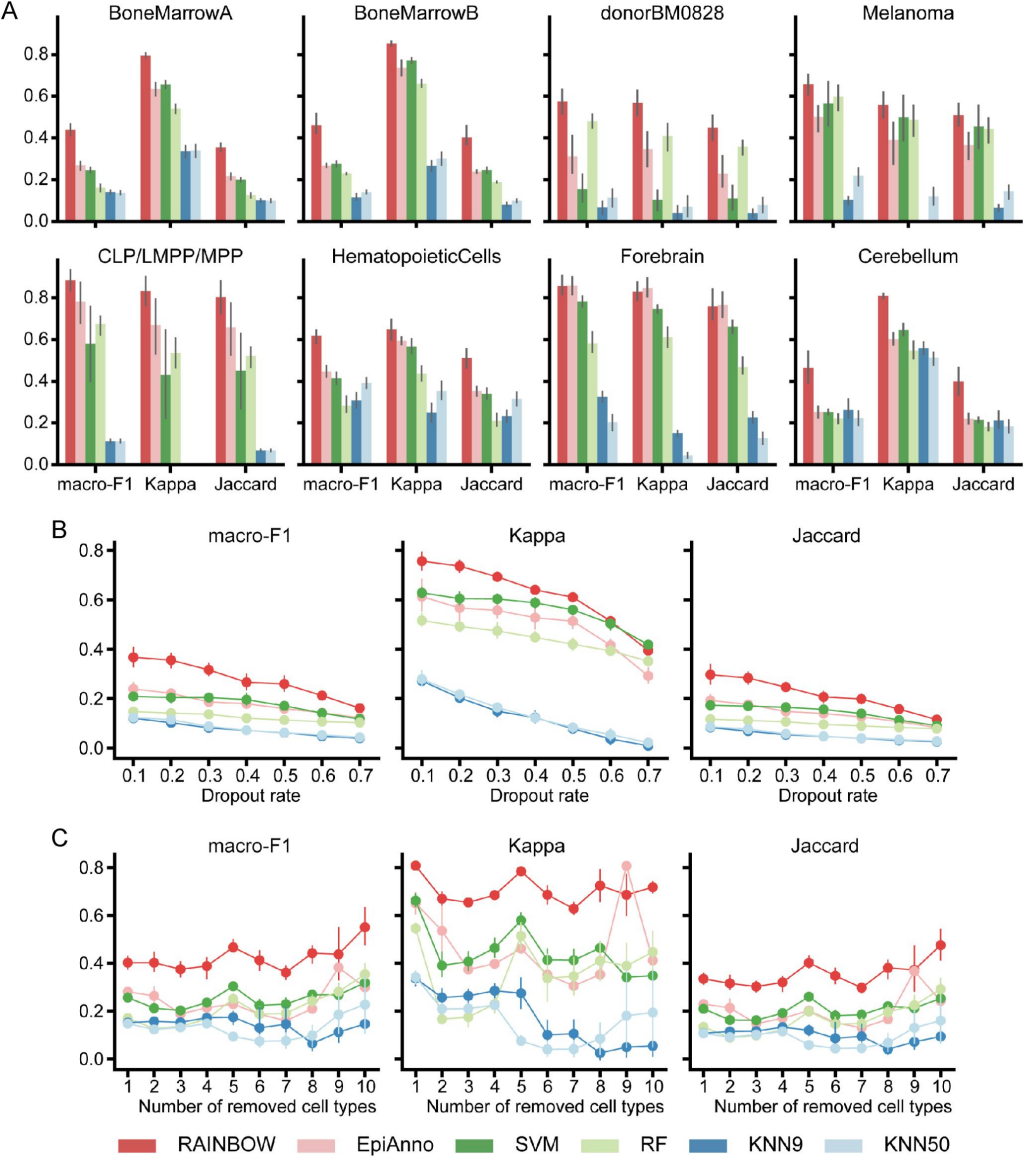
Performance of RAINBOW and other methods. (A) The five‐fold cross‐validation of RAINBOW and baseline methods on the eight datasets. (B) Performance of different methods on the BoneMarrowA dataset with different dropout rates. The dropout rate refers to the probability of setting a non‐zero entry in the data matrix to zero. (C) Performance of different methods on the BoneMarrowA dataset with different numbers of cell types.

As shown in Figure 2A, RAINBOW achieved excellent annotation performance on all the eight datasets. EpiAnno, the current state‐of‐the‐art method, achieved the second‐best overall performance, which is consistent with the benchmark results reported in the original study [4]. As calculated, compared to EpiAnno, RAINBOW increased macro‐F1, Kappa, and Jaccard by 15.79%, 13.41%, and 14.23%, respectively. Furthermore, we performed one‐sided paired Wilcoxon signed‐rank tests to ascertain whether RAINBOW achieved significantly higher metrics on the eight datasets than EpiAnno. The results showed that RAINBOW outperformed EpiAnno across all metrics, yielding statistically significant *p*‐values of 5.110e‐08 for macro‐F1, 1.519e‐07 for Kappa, and 6.859e‐08 for Jaccard. In addition, as the eight datasets have different imbalance degrees, for example, the imbalance degree of donorBM0828 is 0.024 while the imbalance degree of Cerebellum is 0.427, the results also demonstrated the superior robustness of RAINBOW in dealing with imbalanced scCAS datasets. In conclusion, RAINBOW achieves the best annotation performance based on scCAS data.

### 2.3 RAINBOW is robust to the data sparsity

Considering the significant noise and extreme sparsity typically exhibited in scCAS data, it is crucial for a computational method to possess anti‐noise capacity and robustness to data sparsity. To simulate the production of scCAS data, we took the BoneMarrowA dataset as an example, randomly set the non‐zero entries in the data matrix to zero with a probability equivalent to the specified dropout rate and performed five‐fold cross‐validation. The dropout rate was set to range from 10% to 70%.

As shown in Figure 2B, at different levels of data sparsity, RAINBOW consistently exhibited superior performance over other methods by clear margins in most cases. The superiority was particularly striking in the case of dropout rates <40%, where RAINBOW showed an approximately 0.1 improvement across the three metrics comparing to other methods. All the above observations suggest the superior robustness of RAINBOW to the data sparsity.

### 2.4 RAINBOW is robust to the number of cell types

Since the number of cell types varies significantly across different tissues and scCAS samples, we further evaluated the influence of the number of cell types on the annotation performance of different methods. To obtain artificial datasets, we again took the BoneMarrowA dataset as an example and gradually reduced the total number of cell types in the scCAS dataset, ensuring that cell types were randomly removed each time until only two cell types remained.

As shown in Figure 2C, with the total number of cell types in the dataset decreasing, RAINBOW demonstrated significant superiority, especially in scenarios with a larger number of cell types, which is common in practical applications. Furthermore, compared to other methods, RAINBOW was more stable when the total number of cell types varied, particularly in terms of Kappa scores, indicating its heightened robustness. Taken together, RAINBOW is capable of accurately annotating cell types in datasets with varying numbers of cell types, indicating its potential for extensive application in scCAS data analysis.

### 2.5 RAINBOW accurately annotates cells in the inter‐dataset annotation

We next applied RAINBOW for the annotation of newly sequenced data, namely inter‐dataset annotation, which is a more common application scenario. We used four pairs of datasets from Mouse sci‐ATAC‐seq Atlas [18] for demonstration, wherein each pair consisted of two datasets from the same tissue (the whole brain, lung, large intestine, and bone marrow) but different replicates [18]. For each pair, we trained on one dataset and tested on another, and then exchanged the two sets to train and test again.

**FIGURE 3:**
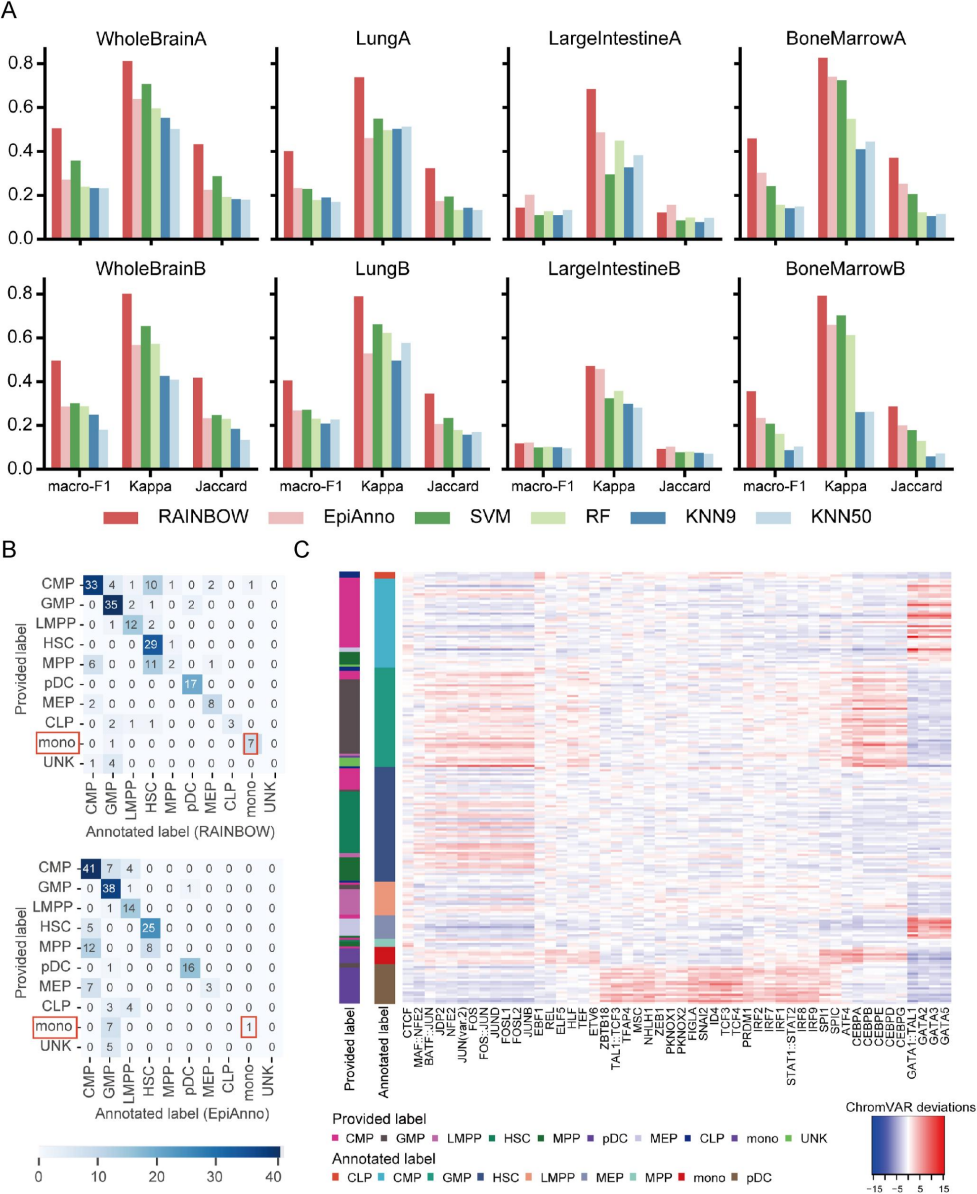
Performance of RAINBOW in the annotation of newly sequenced data and downstream analysis. (A) Performance of RAINBOW and other methods in the annotation of newly sequenced data. The title of each subplot is the name of the training set used in the experiment. (B) The confusion matrix of annotation results obtained from RAINBOW and EpiAnno for the HematopoieticCells dataset. (C) Heatmap of the top 50 most variable TF binding motifs within the peaks specific to each cell type annotated by RAINBOW for the HematopoieticCells dataset. The deviations are calculated by chromVAR.

As shown in Figure 3A, RAINBOW outperformed other methods significantly across all the inter‐dataset experiments, especially in the annotation of whole brain and lung datasets. In comparison to the second‐best method, that is, EpiAnno, RAINBOW improved the average scores of macro‐F1, Kappa, and Jaccard by 12.06%, 17.20%, and 10.55%, respectively. By performing one‐sided paired Wilcoxon signed‐rank tests further, the results demonstrated that RAINBOW is significantly superior to EpiAnno for all the metrics with *p*‐values of 0.020 for macro‐F1, 0.004 for Kappa and 0.020 for Jaccard. The results indicate that RAINBOW enables accurate annotation in newly sequenced data, highlighting the advantages of RAINBOW in practical applications.

### 2.6 Downstream analysis indicates the superior performance of RAINBOW

In the process of cell type annotation, there may be some cells that RAINBOW annotates as the ground‐truth type, while the current state‐of‐the‐art method EpiAnno annotates as different type. This situation may occur either due to the prediction errors of EpiAnno or inaccuracies in the ground‐truth cell type labels. To explore the performance of RAINBOW, we further used the HematopoieticCells dataset as an example to perform downstream analysis. As shown in Figure 3B, RAINBOW annotated cells whose ground‐truth label was monocyte (mono) correctly, while EpiAnno annotated them as granulocyte‐macrophage progenitor (GMP) cells. To verify the accuracy of RAINBOW, we conducted differential accessibility analysis on the cells annotated as monocyte by RAINBOW, and selected the top 1000 peaks with the most significant loadings. We utilized the top peaks to perform Genomic Region Enrichment of Annotation Tool (GREAT) [39] analysis to identify pathways related to the peaks. As shown in Table 1, the pathways that have the top 5 smallest *p*‐values obtained from the binomial test are immune response, cell activation, myeloid leukocyte activation, leukocyte activation, and defense response. The enriched pathways align with the functional characteristics of monocyte cells; monocytes play an important role in immune responses and cellular activation by differentiating into effector cells such as macrophages and dendritic cells, and they also produce various cytokines and signaling molecules that mediate inflammation and immune modulation [40, 41]. Moreover, other significant pathways are related to the positive regulation of immune system process, immune effector process, leukocyte activation involved in immune response, and so on. These processes are consistent with the function of monocyte cells [42, 43, 44]. All the results demonstrate that the cells annotated as monocyte by RAINBOW exhibit the functions that are typical of monocytes, which further indicate that RAINBOW can annotate cell types accurately.

**TABLE 1:** Identified pathways in the GREAT analysis of RAINBOW.

Term name	Binom raw *p*‐value	Binom FDR *Q*‐value
Immune response	2.711E‐26	1.782E‐22
Cell activation	3.705E‐20	1.218E‐16
Myeloid leukocyte activation	2.143E‐18	4.025E‐15
Leukocyte activation	3.545E‐18	5.825E‐15
Defense response	3.746E‐18	5.471E‐15
Positive regulation of immune system process	1.512E‐17	1.988E‐14
Immune effector process	6.434E‐17	7.688E‐14
Leukocyte activation involved in immune response	2.352E‐16	2.378E‐13
Myeloid cell activation involved in immune response	4.295E‐16	3.764E‐13
Cell activation involved in immune response	6.787E‐16	5.576E‐13
Granulocyte activation	2.119E‐15	1.547E‐12
Myeloid leukocyte mediated immunity	3.217E‐15	2.226E‐12
Leukocyte degranulation	5.987E‐15	3.935E‐12
Neutrophil activation	1.581E‐14	9.034E‐12
Neutrophil activation involved in immune response	2.468E‐14	1.352E‐11
Leukocyte mediated immunity	3.207E‐14	1.621E‐11
Response to other organism	3.380E‐14	1.646E‐11

Abbreviations: FDR, false discovery rate; GREAT, Genomic Region Enrichment of Annotation Tool.

Motif enrichment analysis is an important approach in understanding regulatory mechanisms that are specific to certain contexts. We further utilized the HematopoieticCells dataset to demonstrate that the annotation results of RAINBOW can reveal cell type‐specific motifs accurately. We used RAINBOW to annotate cells and selected the 1000 peaks with the smallest *p*‐values following the pipeline [21, 45]. After that, we conducted chromVAR [46] to deduce the enriched motifs of transcription factor (TF) binding in these peaks. The visualization of the top 50 TF binding motifs with the highest variability is presented in Figure 3C. The role of these TFs/motifs in the corresponding cell types is further supported by previous studies. For example, CCAAT Enhancer Binding Protein Beta and CCAAT Enhancer Binding Protein Epsilon are specific to GMP cells [1, 47, 48], GATA1::TAL1, GATA2, and GATA3 are specific to megakaryocyte erythroid progenitor cells [49, 50], SPI1 and ATF4 are specific to monocyte cells [40, 41], and IRF7 and IRF8 are specific to plasmacytoid dendritic cells [51, 52]. All the results show that RAINBOW can reveal motifs that are specific to different cell types for the investigation of gene regulation mechanism.

### 2.7 The effectiveness of incorporating reference data in RAINBOW

The extensive availability of bulk chromatin accessibility data [15, 16, 17] and the increasingly accumulating scCAS data [1, 17, 18, 19, 20] provide ample opportunities to incorporate reference data into the training process. To demonstrate the effectiveness of incorporating reference data on the annotation performance, we further conducted experiments on the eight datasets mentioned in Section 2.2. For each dataset, we incorporated related reference data (see more details in Section 5) into the training process and compared the results with those obtained using only the information from the training set.

**FIGURE 4:**
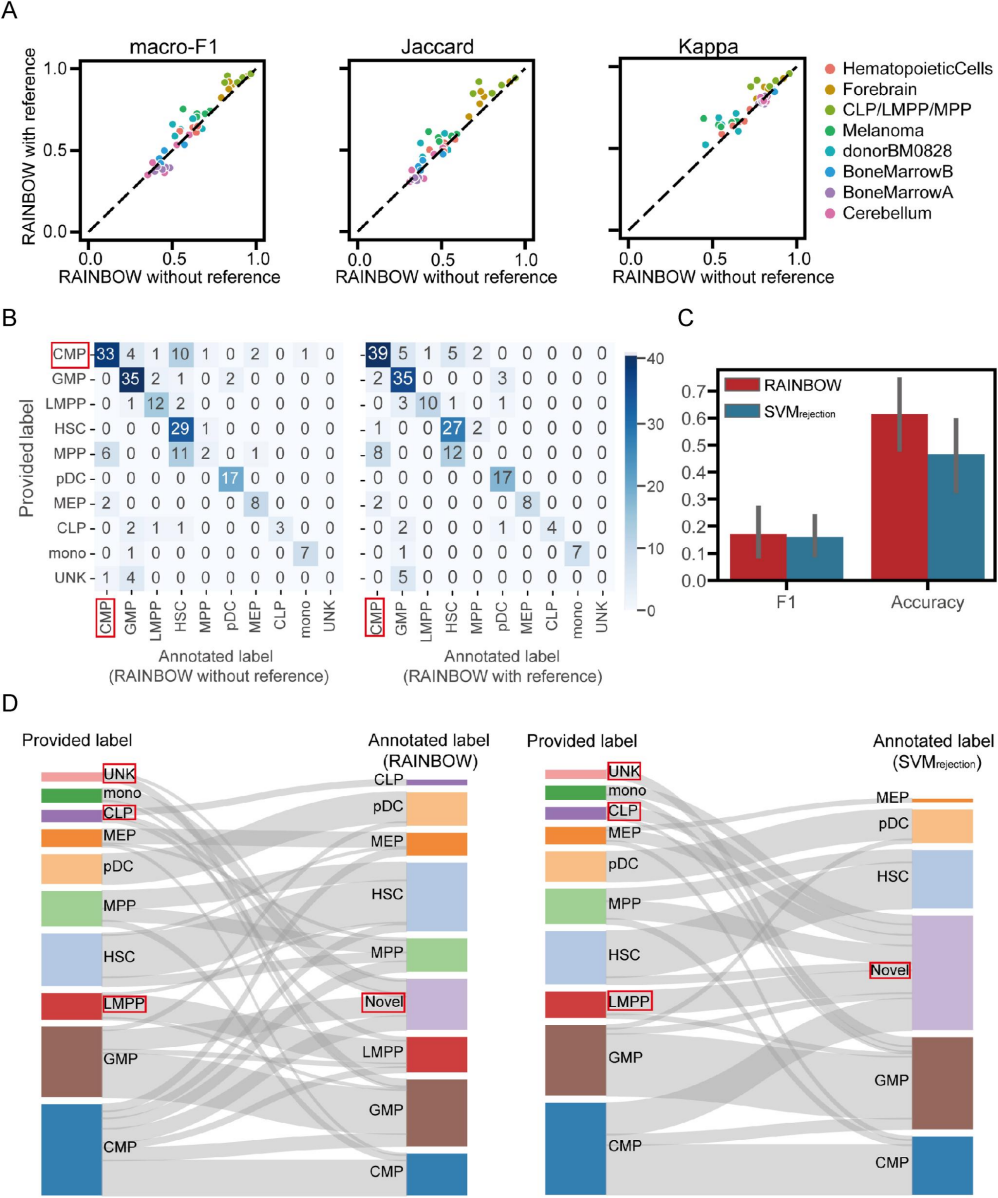
Contribution of incorporating reference data and the identification of the novel type. (A) Performance of RAINBOW without incorporating reference data and RAINBOW with incorporating reference data in the eight datasets. (B) The confusion matrix of annotation results obtained from RAINBOW without incorporating reference data and RAINBOW with incorporating reference data for the HematopoieticCells dataset. (C) Performance of RAINBOW and SVM_rejection_ in the identification of novel type. (D) The Sankey plot of annotation results obtained from RAINBOW and SVM_rejection_ for the HematopoieticCells dataset where UNK cells were treated as the true “novel type.” UNK, unknown.

As presented in Figure 4A, we observed a remarkable enhancement in the annotation performance on most datasets. Especially in the cases of the datasets of CLP/LMPP/MPP, Melanoma, HematopoieticCells, and donorBM0828, incorporating external reference data significantly improved the annotation performance. Furthermore, we performed one‐sided paired Wilcoxon signed‐rank tests to verify the significance of superiority. The results showed that incorporating reference data into RAINBOW significantly improved its performance in all metrics, compared to performance using only the information from the training set, with *p*‐values of 0.008 for macro‐F1, 1.864e‐04 for Kappa, and 8.863e‐04 for Jaccard. Furthermore, we again took HematopoieticCells dataset as an example to illustrate the improvement brought by external reference data. As shown in Figure 4B, RAINBOW incorporated the information from the reference dataset to better annotate CMP cells, which illustrated that this strategy could effectively enhance the heterogeneity of cell types and facilitate more accurate annotation.

### 2.8 RAINBOW can identify novel types accurately

Furthermore, we conducted experiments to verify the superiority of RAINBOW in the identification of novel cell types. Specifically, we used accuracy and F1 as metrics for comparison with SVM_rejection_, as is suggested by the benchmarking study [35], and performed experiments on two datasets, HematopoieticCells and donorBM0828. For each experiment, we removed a cell type from the training set, so that it only appeared in the test set. We treated the removed cell type as the “true” novel type, while other cell types in the test set were considered as non‐novel types. Moreover, novel cell types do not frequently occur and their percentage is often small in practice. Therefore, we only considered cases where the proportion of novel cell type in the test set was <25%.

As shown in Figure 4C, RAINBOW significantly outperformed SVM_rejection_ in the accuracy and F1 scores, thereby validating the effectiveness of RAINBOW in identification of novel cell types. Moreover, RAINBOW can better annotate known types accurately while identifying the novel type. To demonstrate this superiority, we used the experiment where unknown (UNK) cells (with a proportion of 2.45%) in the test set were the “real” novel cell type as an example. As shown in Figure 4D, RAINBOW annotated some UNK cells as novel type, while SVM_rejection_ annotated all UNK cells as GMP type. Furthermore, we note that RAINBOW annotated most CLP cells and LMPP cells accurately, while SVM_rejection_ annotated these cells as novel type. Taken together, RAINBOW can effectively annotate known cell types and identify novel cell type.

## 3 DISCUSSION

With the rapid development of scCAS technology, there is an urgent need for automatic and accurate annotation of cell types. However, existing methods fail to recognize the importance of incorporating external reference data and cannot identify novel cell types, which only exist in the test set. Here, our newly proposed method, RAINBOW, is a reference‐guided automatic annotation method based on contrastive learning, and can identify novel cell types in the test set. With comprehensive experiments on various scCAS datasets, we showed the advantages of RAINBOW over previous state‐of‐the‐art methods, not only in the annotation of known cell types, but also in novel cell type identification. In addition, we demonstrated the robustness of RAINBOW to data sparsity and number of cell types, as well as its advantages in the downstream analysis. Furthermore, RAINBOW provided superior performance in accurately annotating newly sequenced data. All the results indicated the superior performance of RAINBOW in cell type annotation. We anticipate that RAINBOW will offer essential guidance and great assistance in refining cell ontology during scCAS data analysis.

We also describe several avenues for improving RAINBOW. Firstly, we can incorporate the batch information of cells to mitigate the batch effect. Secondly, we can explore different computation methods of the loss function and sampling strategies to design a more effective contrastive learning framework. Finally, we can consider incorporating estimates of the number of cell types [53] to further improve annotation performance or modeling scCAS data using known regulatory elements such as silencers [54] and enhancers [55].

## 4 CONCLUSION

We propose a novel cell type automatic annotation method RAINBOW, based on the contrastive learning framework and with an ability to incorporate external reference data. In addition, RAINBOW possesses the ability to identify the novel cell type effectively, offering researchers a deeper understanding of cellular heterogeneity. With extensive benchmark experiments, we demonstrate that RAINBOW outperforms state‐of‐the‐art methods in both known cell type annotation and novel cell type identification. Moreover, RAINBOW has the potential to reveal novel biological processes and cellular functions.

## 5 MATERIALS AND METHODS

### 5.1 The model of RAINBOW

As shown in Figure 1, the framework of RAINBOW has three main modules, including the training phase, phase of incorporating prior knowledge, and prediction phase. During the training period, given a peak‐by‐cell scCAS count matrix $X\in R^{p\times N}$, RAINBOW first selects peaks with a minimum of one read count in at least 3% of cells in the training set to reduce the noise level, as in other recent scCAS studies [4, 21, 32, 45, 53], where the indices of the selected peaks are saved to perform feature selection for the reference and test set. To normalize the scCAS data matrix, we utilize a technique called TF‐IDF transformation [4, 18, 19, 21, 53]. The importance of peak *i* for cell *j* can be represented by

(1) $$x_{ij}^{\prime }=\frac{x_{ij}}{\sum_{i=1}^{p}x_{ij}}\log\left(\frac{n}{\sum_{j=1}^{n}x_{ij}}\right),$$

and is then normalized via

(2) $$v_{ij}=\frac{x_{ij}^{\prime }}{\sqrt{\sum_{i=1}^{p}{x_{ij}^{\prime }}^{2}}}.$$

Afterward, we can obtain the preprocessed training set ${\tilde{X}}_{\text{train}}$. Then, from the preprocessed training set ${\tilde{X}}_{\text{train}}$, we extract one cell from each cell type randomly to form a subset ${\tilde{X}}_{\text{train}\_a}$ with *k* cells, where *k* is the number of types in the training set. We then repeat this process to obtain another subset ${\tilde{X}}_{\text{train}\_b}.$ These two subsets constitute a training pair $\left({\tilde{X}}_{\text{train}\_a},{\tilde{X}}_{\text{train}\_b}\right)$. In the training pair, cells with the same type $\left({\tilde{x}}_{\text{train}\_a\_i},{\tilde{x}}_{\text{train}\_b\_i}\right)$, where *i* = 1, …, *k*, are regarded as positive instances, while those with different types $\left({\tilde{x}}_{\text{train}\_a\_i},{\tilde{x}}_{\text{train}\_b\_j}\right)$, where *i* ≠ *j* and *i*, *j* = 1, …, *k* are regarded as negative instances. Overall, we generate a total of 10,000 training pairs to train the MLP based on the contrastive learning framework. The model learns latent representations $\left({\tilde{X}}_{\text{train}\_a}^{\prime },{\tilde{X}}_{\text{train}\_b}^{\prime }\right)$ by minimizing the distance between positive instances and maximizing the distance between negative instances. Specifically, the loss function of MLP is defined as follows:

(3) $$\text{loss}=-\log\;\frac{\sum_{i=1}^{k}\exp\left(\text{sim}\;\left(x_{a\_i}^{\prime },x_{b\_i}^{\prime }\right)/\tau \right)}{\sum_{i,j}^{k}1_{\left(i\neq j\right)}\exp\left(\text{sim}\left(x_{a\_i}^{\prime },x_{b\_j}^{\prime }\right)/\tau \right)}$$

(4) $$\text{sim}\left(u,v\right)=\frac{u^{T}v}{∥u∥\cdot ∥v∥},$$

where $x_{a\_i}^{\prime }$ and $x_{b\_i}^{\prime }$ are the *i*th cells in ${\tilde{X}}_{\text{train}\_a}^{\prime }$ and ${\tilde{X}}_{\text{train}\_b}^{\prime }$, respectively; *τ* is the temperature coefficient, a hyperparameter that controls the difficulty of distinguishing between positive and negative instances in a sample pair; 1_(*i* ≠ *j*)_ is the indicate function. From the loss function, it can also be seen that contrastive learning focuses on learning the common features among instances of the same type and highlighting the distinctions between instances of different types, thereby enhancing the heterogeneity of cell types and facilitating more accurate annotation.

At the stage of incorporating prior knowledge, RAINBOW offers two options: either incorporating external reference data, or not. If there is no reference data, only the information from the training set is utilized by RAINBOW as prior knowledge. Within each cell type of the preprocessed training set ${\tilde{X}}_{\text{train}},$ we carry out 500 repeated random samplings with replacement to obtain a sample count matrix ${\tilde{X}}_{\text{prior}\_k}$. We then repeat the process 1000 times to obtain 1000 sample count matrices. By computing the mean of each sample count matrix ${\tilde{X}}_{\text{prior}\_k}$, we can construct the prior sample ${\tilde{X}}_{\text{prior}}$. Afterward, we perform PCA (with a total of 100 principal components) on the prior sample ${\tilde{X}}_{\text{prior}}$ and utilize the mapping matrix obtained to initialize the weights connecting the first to the second layer of MLP. On the other hand, if an external reference set is available during the training process, we first perform feature selection using the saved peak indices from the training set and TF‐IDF transformation consistent with the training phase on the reference set. Then we perform PCA (with a total of 100 principal components) to the count matrix of the preprocessed reference set. Subsequently, we combine the projection matrix of the reference set with that of the training set to serve as the initialization of the weights connecting the first to the second layer of the MLP, which allows us to utilize prior knowledge from the external reference set into the training process. It should be noted that the number of neurons in the second layer of MLP will change when incorporating reference data. Specifically, if there is no reference data, the second layer contains two sets of neurons: one set with 128 neurons, whose weights connecting to the first layer are randomly initialized, and another set with 100 neurons, whose weights connecting to the first layer are initialized using the weights of PCA derived from the training set. If we incorporate reference data, we will add a third set of neurons to the second layer, and the weights connecting these neurons to the first layer will be initialized using the weights of PCA derived from the reference set.

In the predicting phase, following feature selection with the saved peak indices from the training set, and TF‐IDF transformation consistent with the training phase, the preprocessed test set is fed into the trained MLP to obtain its low‐dimensional representation. For a cell in the test set, we calculate its cosine similarity with respect to each cell in the training set based on their latent representations. By computing the mean cosine similarity within each type, we select the cell type that corresponds to the maximum cosine similarity value as the label.

Notably, RAINBOW offers the capacity to identify novel cell types, which only exist in the test set. Herein, for each cell in the test set, the entropy of cosine similarity is viewed as a metric to measure prediction uncertainty. A higher entropy value indicates greater uncertainty in the prediction. For the preprocessed test set, we perform Leiden clustering [34] on the cell embeddings obtained from PCA. To better distinguish between novel and known types, the number of clusters is specified as follows:

(5) $$n_{\text{cluster}}=\alpha \times n_{\text{type}},$$

where $n_{type}$ is the unique number of cell type labels in the training set and *α* is specified as 1.5. We compute the entropy of each cell and then annotate cells in clusters with mean entropy values >0.7 as novel type.

### 5.2 Baseline methods

As mentioned in Section 1, there are two schools of cell annotation methods. However, it is hard and not feasible to compare RAINBOW with methods of the first group, as they are manual and subjective and follow fundamentally different principles from methods of the second group. Hence, we only compared the performance of methods of the second group, as suggested by benchmarking studies for cell type annotation methods [4, 31, 35].

We compared the performance of RAINBOW with 5 baseline methods when no novel cell types exist in the test set. Among these methods, EpiAnno is the first and the state‐of‐the‐art cell type annotation method tailored for scCAS data [4], and the other methods are conventional machine learning methods (SVM with a linear kernel [12], RF [13], KNN [14] with K of 9 and 50, respectively) as suggested by recent benchmark studies [35, 56]. To evaluate the performance of identifying novel cell types, since there were no existing methods specifically designed for scCAS data, we adopted SVM_rejection_ as the baseline method suggested by the benchmark study [35]. SVM_rejection_ assigns a cell to novel cell type if the probability belonging to the most probable cell type is <0.7. The threshold of 0.7 was utilized in the benchmark study [35]. We obtained the source code to implement EpiAnno from [4]. Source codes for implementing the machine learning methods were obtained from [35]. None of the supervised methods mentioned above require any additional information apart from the annotated cell labels in the training sets. We benchmarked the performance of different methods following their tutorial and using their default parameters. The benchmark experiments were conducted on a machine with two Intel Xeon Platinum 8375C CPUs, two NVIDIA RTX A6000 GPUs, and 256 GB of RAM.

### 5.3 Data collection

We collected 14 datasets that were produced using different protocols and involved various species, sizes, dimensions, cell states, levels of sparsity, numbers of batches, and imbalances in cell types for comprehensive evaluation.

Firstly, we collected human HematopoieticCells labeled as donor BM0828 from a bone marrow single‐cell ATACseq (Fluidigm C1) dataset [1], and designated this dataset as donorBM0828. Subsequently, we collected a subset of bone marrow cells consisting of CLPs, LMPPs, and MPPs from 2 donors, and designated this dataset as CLP/LMPP/MPP. Moreover, we collected the dataset HematopoieticCells, an entire dataset of bone marrow cells containing 10 differentiating cell types from 7 donors from the same study [1]. The above three datasets offer cell type labels produced through fluorescent‐activated cell sorting. Furthermore, to evaluate different methods for annotating the differentiating cell types, we collected a dataset that consists of cells in time series after the knockdown of SOX10 in melanoma cell lines of two short‐term patient cultures [36], and we referred it as Melanoma.

Additionally, to assess the generalizability of our findings across different species and data sources, we collected Forebrain, which was derived from mouse forebrain by a single nucleus ATAC‐seq and was used to evaluate the performance of different methods on cells derived from complicated tissue [20]. scCAS experiments usually generate data with varying numbers of cells for different cell types, and often with high levels of cell‐type imbalance, so to evaluate how imbalanced degrees of different cell types affect the experiment results, we collected 9 datasets from Mouse sci‐ATAC‐seq Atlas [19]. All these datasets were generated by a combinatorial indexing assay (sci‐ATAC‐seq), consisting of both differentiated and differentiating tissues (two replicates of bone marrow, two replicates of large intestine, two replicates of lung, two replicates of whole brain, and cerebellum), with different levels of sparsity [19].

We utilized the cell type labels annotated in the original studies of the datasets as the ground‐truth labels for evaluation. All the cell type labels of datasets were annotated via performing unsupervised cell clustering and then assigning the putative cell‐type label to each cluster manually (e.g., datasets of Forebrain), or by experimental fluorescent activated cell sorting (e.g., datasets of donorBM0828). The cell‐type labels were considered reasonable and have been used to assess unsupervised cell clustering performance and supervised cell type annotation performance [3, 4, 21, 32, 45, 57, 58].

Specifically, we divided all of our data into two equal parts. One of the halves was employed for five‐fold cross‐validation, while the other half was designated as an “external reference set” to incorporate into the model. Notably, given that the baseline methods could not incorporate reference data, to ensure fairness, we did not incorporate the other half of the “external reference set” when comparing with baseline methods. The “external reference sets” were only utilized in the Section 2.7 wherein we verified the effectiveness of incorporating reference data.

As presented in Table 2, the summary of 14 scCAS datasets collected. The degree of imbalance in the dataset is defined by calculating the normalized entropy of the distribution of cell‐type sizes, which can be calculated as follows:

(6) $$I=1+\frac{1}{\log\;K}\sum_{k=1}^{K}\frac{N_{k}}{N}\log\frac{N_{k}}{N},$$

where *K* is the total number of ground‐truth cell types in the dataset, *N*_*k*_ is the number of cells of the *k*th cell type, and *N* is the total number of cells in the dataset. The degree of imbalance ranges from 0 to 1, wherein a greater degree indicates that this dataset is more unbalanced. For example, if all cells belong to a single cell type, the degree will be one, while if all the cell types have equal cell counts, the degree will be zero. We defined the sparsity of a dataset as the ratio of zero elements in the scCAS count matrix. The proportion of major type is defined as the cell proportion of the type with the most cells.

**TABLE 2:** Summary of datasets used in this study.

Dataset	No. of cells	No. of peaks	No. of batches	No. of cell types	Species	Cell state	Sparsity	Imbalance degree	Proportion of major type
donorBM0828	533	455,057	1	7	*Homo sapiens*	Differentiating	0.991	0.024	0.240
CLP/LMPP/MPP	380	455,057	2	3	*Homo sapiens*	Differentiating	0.993	0.038	0.421
HematopoieticCells	2034	455,057	7	10	*Homo sapiens*	Differentiating	0.990	0.106	0.247
Melanoma	598	78,661	2	4	*Homo sapiens*	Differentiating	0.954	0.001	0.266
Forebrain	2088	140,102	1	5	*Mus musculus*	Differentiated	0.990	0.207	0.515
BoneMarrowA	4370	436,206	1	18	*Mus musculus*	Differentiating	0.992	0.399	0.367
BoneMarrowB	4033	436,206	1	15	*Mus musculus*	Differentiating	0.991	0.397	0.402
LargeIntestineA	2281	436,206	1	18	*Mus musculus*	Differentiated	0.990	0.572	0.561
LargeIntestineB	4805	436,206	1	18	*Mus musculus*	Differentiated	0.990	0.725	0.696
LungA	5122	436,206	1	22	*Mus musculus*	Differentiated	0.992	0.274	0.250
LungB	4874	436,206	1	25	*Mus musculus*	Differentiated	0.991	0.262	0.216
WholeBrainA	5494	436,206	1	21	*Mus musculus*	Differentiated	0.985	0.295	0.348
WholeBrainB	3272	436,206	1	20	*Mus musculus*	Differentiated	0.983	0.273	0.333
Cerebellum	2278	436,206	1	20	*Mus musculus*	Differentiated	0.992	0.427	0.479

Abbreviations: CLP, common lymphoid progenitor; LMPP, lymphoid‐primed multipotent progenitor; MPP, multipotent progenitor.

### 5.4 Model evaluation

For the scenario in which there are no novel types in the test set, we benchmarked the annotation performance using three metrics, macro‐F1 score, Cohen’s kappa value (Kappa) [37] and Jaccard, as suggested by recent studies [4, 31, 38]. The macro‐F1 score is suitable for multi‐class classification problems and is not influenced by imbalanced cell types; it can be calculated as follows:

(7) $${\text{F}1}_{i}=2\times \frac{{\text{recall}}_{i}\times \text{precisio}n_{i}}{{\text{recall}}_{i}+\text{precisio}n_{i}}$$

(8) $$\text{macro}-\text{F}1=\frac{1}{K}\sum_{i=1}^{K}{\text{F}1}_{i},$$

where precision_*i*_ represents the proportion of cells correctly annotated as the *i*th cell type to the total number of cells annotated as that cell type, while recall_*i*_ represents the proportion of cells correctly annotated as the *i*th cell type to the total number of cells of that cell type. Macro‐F1 score is the arithmetic mean of the F1 scores for all cell types, and *K* is the number of cell types in the dataset. Kappa takes the possibility of the agreement occurring by chance into account, which can be calculated as follows:

(9) $$\kappa =\frac{\text{acc}-p_{e}}{1-p_{e}},$$

where acc represents the accuracy of annotation and *p*_*e*_ is the hypothetical probability of chance variable calculated as follows:

(10) $$p_{e}=\frac{1}{N^{2}}\sum_{k}a_{k}b_{k},$$

where *N* is the total number of cells, *a*_*k*_ and *b*_*k*_ are the number of the *k*th cell type in the classification result and the real dataset, respectively. Jaccard similarity coefficient is commonly used to evaluate the similarity between the classification results and the true results, with the following formula:

(11) $$\text{Jaccard}=\frac{\left|A⋂B\right|}{\left|A⋃B\right|},$$

where A is the set of predicted labels and B is the set of true labels. The closer the Jaccard score is to 1, the more similar the predicted labels are to the true labels.

For the binary classification where there exist novel types in the test set, we adopted F1 score and accuracy as the metrics to evaluate the performance. F1 score is calculated using Equation (7), and accuracy can be calculated as follows:

(12) $$\text{accuracy}=\frac{t}{N},$$

where *t* is the number of cells for which the annotated result is consistent with the true label, *N* is the total number of cells.

### 5.5 Downstream analysis

#### 5.5.1 GREAT analysis

After obtaining the annotation results, we selected the top 1000 peaks with the most significant loadings in cells annotated as monocyte by RAINBOW and then submitted the peaks to the GREAT server [39] using the default settings in order to identify pathways related to the peaks, and thereby obtaining functional understanding for the cells which are annotated as monocyte by RAINBOW.

#### 5.5.2 Motif enrichment analysis

As suggested by the pipeline [21, 45, 46], we performed motif enrichment analysis. With the annotated cell labels obtained from RAINBOW, we identified the cluster‐specific peaks by implementing the hypothesis testing procedure in scABC [45]. The top 1000 peaks with the smallest *p*‐values for each cluster were selected and subjected to the analysis of TF binding motif enrichment using chromVAR [46].

## AUTHOR CONTRIBUTIONS

**Siyu Li**: Methodology; investigation; writing – original draft preparation; writing – review & editing. **Songming Tang**: Validation. **Yunchang Wang**: Methodology. **Sijie Li**: Validation. **Yuhang Jia**: Visualization. **Shengquan Chen**: Conceptualization; funding acquisition; supervision; writing – review & editing.

## CONFLICT OF INTEREST STATEMENT

The authors Siyu Li, Songming Tang, Yunchang Wang, Sijie Li, Yuhang Jia, and Shengquan Chen declare that they have no conflicts of interest.

## ETHICS STATEMENT

This article does not contain any studies with human or animal materials performed by any of the authors.
